# Molecular Energy Landscapes of Hardware-Efficient Ansätze in Quantum Computing

**DOI:** 10.1021/acs.jctc.2c01057

**Published:** 2023-02-07

**Authors:** Boy Choy, David J. Wales

**Affiliations:** † School of Chemistry, Chemical Engineering and Biotechnology, Nanyang Technological University, Block N1.2, B3-13, 62 Nanyang Drive, Singapore 637459; ‡ Yusuf Hamied Department of Chemistry, University of Cambridge, Lensfield Road, Cambridge CB2 1EW, United Kingdom

## Abstract

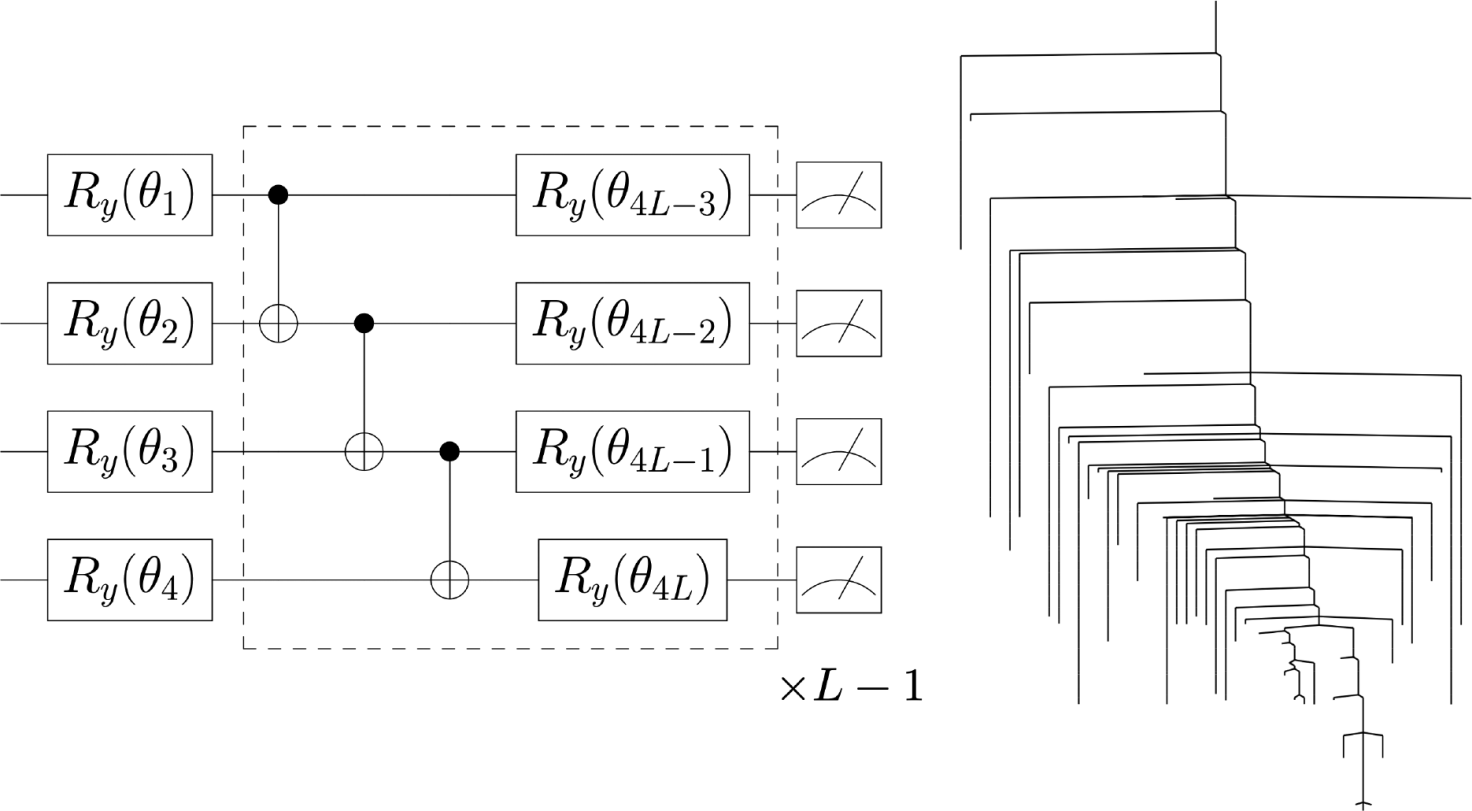

Rapid advances in quantum computing have opened up new opportunities for solving the central electronic structure problem in computational chemistry. In the noisy intermediate-scale quantum (NISQ) era, where qubit coherence times are limited, it is essential to exploit quantum algorithms with sufficiently short quantum circuits to maximize qubit efficiency. The procedural construction of hardware-efficient ansätze provides one approach to design such circuits. However, refining the accuracy of the global minimum by increasing circuit depth may lead to a proliferation of local minima that hinders global optimization. To investigate this phenomenon, we explore the energy landscapes of hardware-efficient circuits to identify ground-state energies of the hydrogen, lithium hydride, and beryllium hydride molecules. We also propose a simple dimensionality reduction procedure that reduces quantum gate depth while retaining high accuracy for the global minimum, simplifying the energy landscape, and hence speeding up optimization from both software and hardware perspectives.

## 1 Introduction

The intractability of the many-body problem continues to pose enormous challenges in finding the ground-state energy of molecules for traditional classical computing.^1,2^ Hence, the potential of quantum computing has sparked considerable excitement in tackling the exponential scaling associated with the electronic structure problem,^3^ potentially advancing the discovery of novel drugs^4^ and catalysts^5^*in silico*. However, the inherent limitations associated with current noisy intermediate-scale quantum (NISQ) devices, mainly short qubit decoherence times and low error correction,^6^ preclude the feasible implementation of exact algorithms such as quantum phase estimation (QPE).^7^ Instead, hybrid classical–quantum algorithms more resilient to quantum noise, such as the variational quantum eigensolver (VQE),^8^ have been successfully employed in actual quantum hardware in calculating the ground-state energy of various molecules.^9^ This approach is expected to prevail until more fault-tolerant quantum devices supporting a larger number of implementable qubits are developed.^10^

As the VQE algorithm seeks to variationally constrain the upper bound for the ground-state energy of a target molecule,^11^ choosing a good circuit ansatz for the approximation is crucial. Several approaches exist in the selection of ansätze: one method involves utilizing the unitary coupled cluster (UCC) ansatz that typically employs excitations within the electronic structure of the molecule, where single and double excitations are most commonly used.^12^ However, the number of quantum gates required to implement the traditional UCC ansatz typically grows rapidly as the complexity of the target molecule increases.^13^ Although various methods have been developed in refining the UCC ansatz to curtail the number of excitation operators that need to be implemented, for example, via the ADAPT-VQE algorithm that selects cluster operators based on their contribution to the overall gradient function,^14^ their computational implementation via standardized quantum gates within quantum circuits onto contemporary quantum computers still remains difficult in practice.^15^ Furthermore, it has also been suggested that the ADAPT-VQE algorithm requires a greater number of measurements to be made on a quantum computer compared to standard VQE.^16^

A less demanding approach, which is the main focus of this study, is to employ hardware-efficient ansätze that aim to improve the convergence of the ground-state energy of a given molecule via the progressive introduction of more parametrized and entangling quantum gate layers. This simple, yet effective, strategy has been successfully implemented in quantum hardware to find the ground-state energies of hydrogen, lithium hydride, and beryllium hydride.^17^ However, as the primary goal of the hardware-efficient ansatz is to locate the global minimum rather than preserve the symmetries of the molecule, a large number of gate layers may be needed to achieve convergence. This issue becomes increasingly problematic for larger systems requiring a greater number of qubits due to the barren plateau problem, where in the presence of noise the gradients decrease exponentially as the number of qubits and circuit layers increases.^18^ Furthermore, the inclusion of more circuit layers may lead to the introduction of numerous local minima, making global optimization more difficult. Finally, the increase in the number of implemented quantum gates may result in compounding of gate noise arising from depolarization, thermal relaxation, and dephasing errors.^19^

The employment of basin-hopping methods to study energy landscapes arising from quantum computing is relatively scant, with a notable example being the enhancement of Grover’s algorithm by means of a quantum basin-hopper.^25^ We therefore seek to advance the exploration of hardware-efficient ansätze in computing the ground-state energies of hydrogen, lithium hydride, and beryllium hydride via the VQE algorithm using the principles of energy landscape theory and associated computational tools, which have been employed successfully in treating relatively high-dimensional molecular systems.^26−29^ By developing the tools necessary to analyze the solution landscapes of hardware-efficient ansätze in greater detail, we provide greater insight into the proliferation of local minima and other stationary points arising from VQE optimization,^30^ and thus design new strategies to mitigate their occurrence and ensure an efficient search for the global minimum. We also devise a simple deparameterization procedure that aims to reduce the parameter space of hardware-efficient ansätze, while still retaining the accuracy of the global minimum, thus simultaneously accelerating global optimization and reducing quantum gate noise.

## 2 Methodology

**Figure 1:**
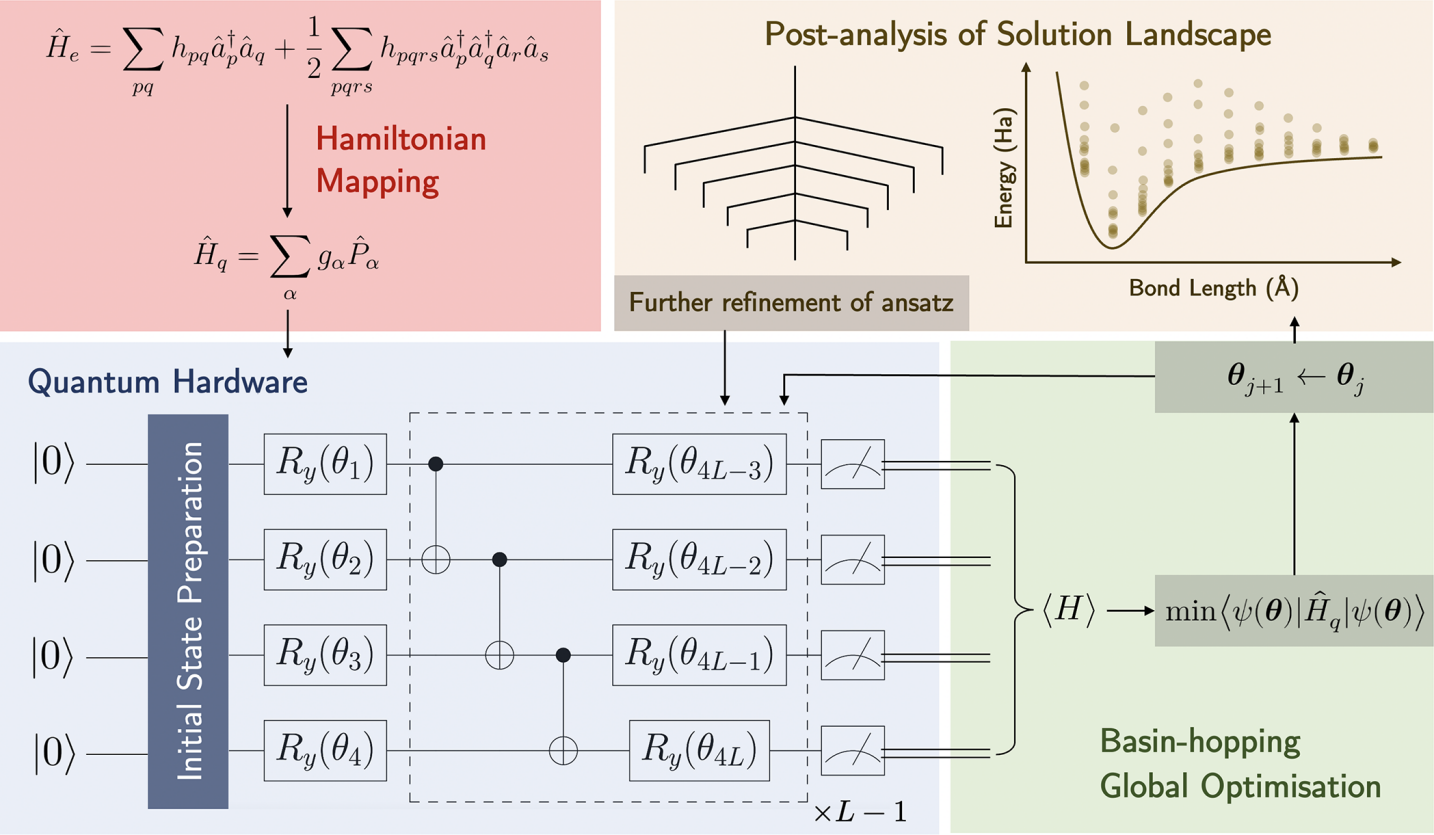
Schematic of the methodology employed in this study. After parity transforming the electronic Hamiltonian of a molecule *Ĥ*_*e*_ into its qubit equivalent *Ĥ*_*q*_, the ground-state energy is approximated via the VQE algorithm by first evolving an initial state |ψ(0)⟩ with a suitable hardware-efficient ansatz for unitary operator *Û*(**θ**) to give the final state |ψ(**θ**)⟩. The expectation value ⟨ψ(**θ**)|*Ĥ*_*q*_|ψ(**θ**)⟩ is then calculated on a classical computer and minimized. Basin-hopping global optimization^20−22^ is then employed to propose steps in parameter space. The VQE algorithm is iterated between the quantum–classical computer interface until a suitable convergence criterion is met, and this process is repeated for all required bond lengths of the molecule, yielding the potential energy surface and stationary points relevant to the circuit ansatz. Finding transition states between various minima allows for visualization of the ansatz solution landscape using disconnectivity graphs.^23,24^ Subsequent deployment of the dimensionality reduction, or deparameterization, procedure can further refine the circuit ansatz by reducing its parameter depth while retaining the same global minimum.

Figure 1 outlines the integration of the energy landscape exploration program packages GMIN,^31^ OPTIM,^32^ and PATHSAMPLE^33^ with the base VQE algorithm used in approximating the ground-state energy of a given molecule for a particular geometry. The general methodology begins by first considering the electronic Hamiltonian *Ĥ*_*e*_ of the molecule in its second quantized form
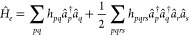
1where *h*_*pq*_ and *h*_*pqrs*_ are the one- and two-body integrals and *â*^†^ and *â* are the fermionic creation and annihilation operators, respectively. *Ĥ*_*e*_ is then isospectrally mapped to a suitable qubit Hamiltonian *Ĥ*_*q*_. We employ parity transformation with two-qubit reduction^34^ via the PySCF package to yield a linear combination of Pauli strings *P̂*_α_ with coefficients *g*_α_, thus compacting the number of encoded qubits *N*(35) (although one could also utilize other standardized mappings such as Jordan–Wigner or Bravyi–Kitaev^36^)
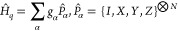
2The lowest eigenvalue of *Ĥ*_*q*_, and thus the ground-state electronic energy of the molecule, *E*_0_, can then be variationally approximated with the VQE algorithm. We evolve an initial Hartree–Fock state |ψ(0)⟩ with a suitable hardware-efficient ansatz for unitary operator *Û*(**θ**) and *L* layers by means of a quantum computer
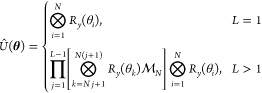
3with the *R*_*y*_-parametrized rotation gates taking on the standard form
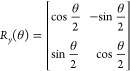
4Each *L* component is composed of a parametric layer  equipped with *R*_*y*_ rotation gates for each qubit and a linear entangling layer , where *CNOT* gates are arranged in a linear fashion from the first to the *N*th qubit
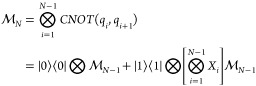
5setting  for the recursive case. The parametric *R*_*y*_-linear entangling circuit has been used successfully as a shallow yet effective hardware-efficient ansatz in finding the ground-state energies of various molecules via VQE.^37,38^

The final evolved state |ψ(**θ**)⟩ = *Û*(**θ**)|ψ(0)⟩ can be used to evaluate the expectation value *E*(**θ**) on a classical computer, which the variational principle implies must be greater than or equal to *E*_0_
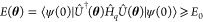
6Thus, for a given convergence criterion, as the number of layers of the hardware-efficient ansatz increases, it is expected that the increase in parametric expression from  and entangling power from  would allow for a better approximation of *E*_0_ up to a certain minimum circuit layer depth *L*_min_. For a given iteration number *k* with angular parameters **θ**(*k*) = {θ_1_, θ_2_, . . . , θ_μ_, . . . , θ_*LN*_}, we employ basin-hopping^20−22^ to find the global minimum of *E_k_* using the GMIN program with local minima characterized by a limited-memory Broyden–Fletcher–Goldfarb–Shanno (L-BFGS) algorithm.^39^ During basin-hopping global optimization, the energy of the succeeding iteration, *E*_*k*+1_, is accepted via the Metropolis criterion, i.e., if *E*_*k*+1_ < *E*_*k*_ or with probability exp(−(*E*_*k*+1_ – *E*_*k*_)/*T*) otherwise, where *T* is an effective temperature in units of energy.^20−22^ If *E*_*k*+1_ is not accepted then a random perturbation of up to 1.0 rad is performed on **θ**(*k*) and the optimization proceeds as usual. The parameter-shift rule is used to compute the analytic gradients for each angular parameter, since the only parametrized gates are localized single-qubit *R*_*y*_ gates with two unique eigenvalues^40^
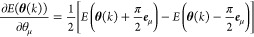
7where ***e***_μ_ is the unit vector of parameter θ_μ_. Subsequently, the updated parameters of iteration *k* + 1 are relayed back into the quantum computer for the computation of a new *E_k_*_+1_. This process between the quantum–classical computer interface continues until a suitable convergence criterion is reached. For this study, 5000 basin-hopping steps with a root-mean-squared (RMS) gradient convergence criterion of 5 × 10^–10^ au and a reduced temperature of 1.0 au were used to perform global optimization via GMIN for the H_2_, LiH, and BeH_2_ molecules (see section 1 of the Supporting Information for a further description of the optimization parameters used in all GMIN runs). For the LiH and BeH_2_ examples we employed a reduced active space, as our main focus is to characterize and contrast the energy landscapes arising from the *Ĥ*_*q*_ operators of LiH and BeH_2_ where the former is much more compact than the latter owing to its greater number of terms, despite encoding fewer qubits. However, basin-hopping methods can certainly be applied to molecules in the full-space configuration. Basin-hopping runs generally sample a number of minima in addition to the global minimum. To define the landscape for *E*(**θ**) we employ the OPTIM program to locate transition states and characterize the corresponding pathways that connect pairs of minima. To classify each stationary point as a minimum, transition state, or higher order saddle point, the *LN* × *LN* Hessian of the circuit ansatz **H** was constructed by iterating the parameter-shift rule again from eq 7 over another parameter θ_*ν*_ for Hessian element *H*_μν_
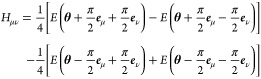
8followed by diagonalization of **H** to determine the number of negative eigenvalues *V*. The local minima, i.e., stationary points with zero negative eigenvalues for their respective Hessians, can then be filtered from other stationary points. Double-ended connection runs between pairs of minima begin with a doubly-nudged^41^ elastic band (DNEB) calculation, where candidate transition states are further refined with hybrid eigenvector following.^42−45^ Here, the convergence condition for the Rayleigh–Ritz calculation of the smallest nonzero Hessian eigenvalue was set at 10^–5^ au. To compare the distance between two fully evolved states ψ(**θ**(γ)) and ψ(**θ**(δ)) with different rotation parameters, the state vector overlap *S*_*γδ*_ was used as a metric
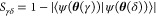
9The energy landscape corresponding to the database of local minima and transition states can be visualized using disconnectivity graphs,^23,24^ as discussed below.

In some cases the Hamiltonian *Ĥ*_*q*_ is sufficiently sparse that full parametrization of all *R*_*y*_ gates in a circuit ansatz with depth *L*_min_ (or higher) is not necessary to produce a global minimum with an accurate energy. Thus, to reduce the parameter depth even further, a heuristic deparameterization procedure can be adopted. The most straightforward implementation is as follows: first, a parametrized *R*_*y*_ gate is selected and frozen by assigning the gate a fixed rotation value. A rotation amplitude of zero is typically sought, although other standardized values such as ±π/2 or ±π can also be used. The former choice is most desirable for various reasons: it allows for the reduction of the *R*_*y*_ gate into a virtual identity gate that does not need to be implemented in practice, thus eliminating any associated quantum gate noise. In terms of software, the computational cost of optimizing the parameter associated with that gate is also saved. Finally, by aiming to find as many virtual identity gates as possible, we can construct quantum circuits with more degrees of freedom for circuit transpilation, thus potentially allowing for more efficient mapping of the quantum circuit onto actual quantum hardware and subsequently reducing its effective depth.^46^ The VQE algorithm is then performed for the reformulated circuit ansatz. If the same global minimum energy for all required bond lengths can be achieved as for the original circuit then the deparameterization of that *R*_*y*_ gate is maintained; otherwise, it is reparameterized. Another *R*_*y*_ gate is then chosen and deparameterized, and this process continues until no more *R*_*y*_ gates can be frozen without degrading the accuracy, thus yielding a refined circuit that is suitable for all molecular geometries sampled.

If carried out in this manner, the maximum number of runs in implementing the deparameterization procedure would be *LN* – 2, since for a linear ansatz as in eq 3, the first *R*_*y*_ gate in sequence is the most important in varying the evolved state and is thus set to be parametrized by default. However, it is practically more efficient to compare similar rotation amplitudes of global minima obtained for different bond lengths, simultaneously picking out multiple *R*_*y*_ gates that conform to the standardized values of {0, ±π/2, ±π} to be deparameterized. Conversely, the *R*_*y*_ gates with more variable rotation amplitudes across multiple molecular geometries are chosen to remain parametrized throughout the procedure. Overall, the flexible designation of multiple parametrized and deparameterized gates within the same run via comparison of amplitudes across sampled bond lengths reduces the overall number of times the deparameterization procedure needs to be implemented, thus providing substantial savings to the additional hardware and software computation costs incurred from implementing the deparameterization procedure.

We also find that reducing the number of parameters may lead to simplification of the energy landscape for the refined ansatz, reducing the number of minima. However, new stationary points, arising from the truncation of the stationary points in the original circuit ansatz, can also appear. Hence, it is important to compare the refined and unrefined ansätze to determine if the desired energy landscape simplification has been achieved.

## 3 Results and Discussion

### 3.1 Hydrogen

**Figure 2:**
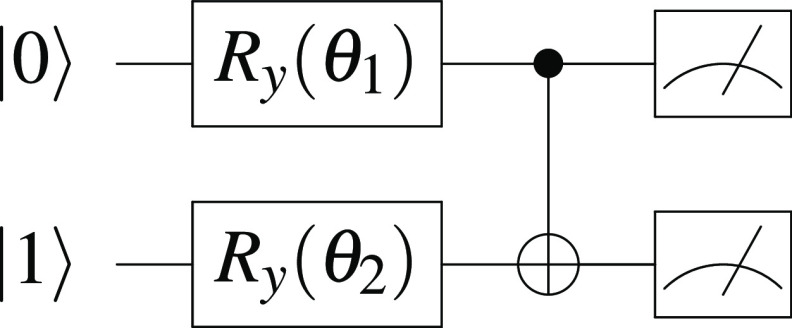
Two-qubit circuit ansatz of H_2_ used in calculating the ground-state energy.

**Figure 3:**
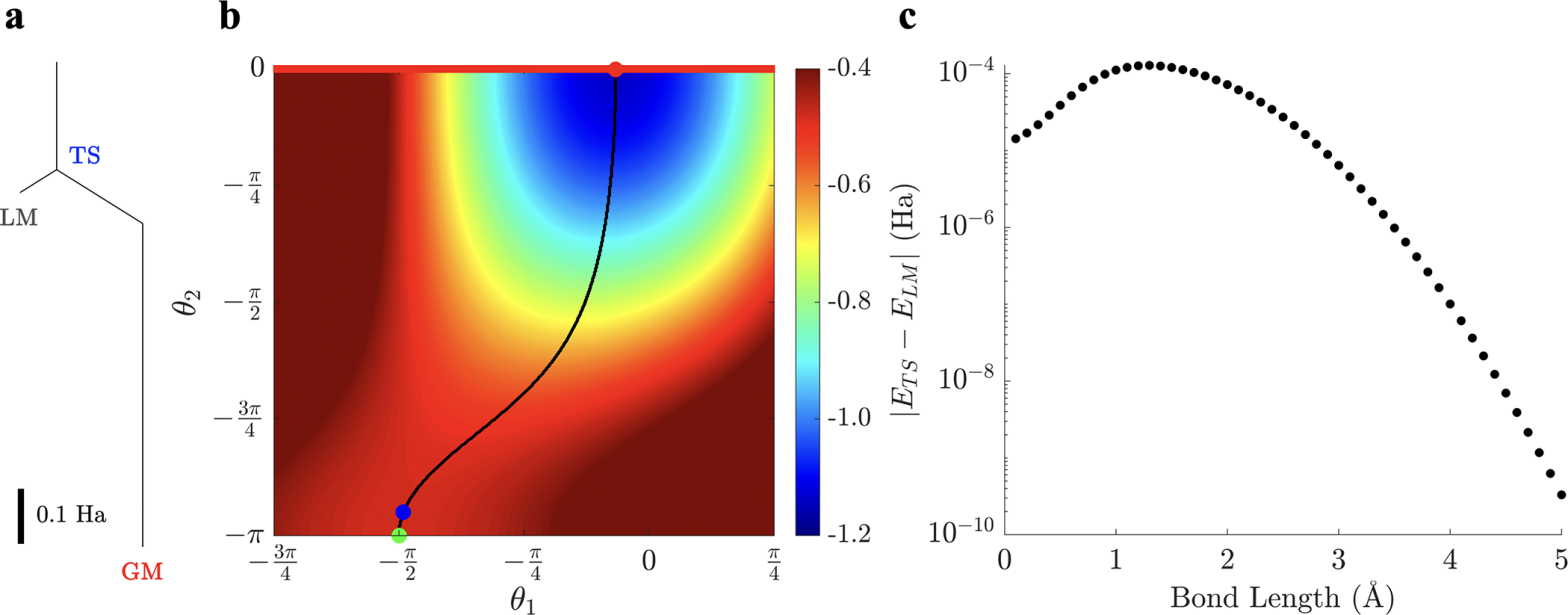
(a) Disconnectivity graph of the circuit ansatz connecting the global minimum (GM, in red), the local minimum (LM, in green), and the transition state between them (TS, in blue) for a H–H bond length of 0.7 Å. (b) Three-dimensional electronic energy landscape of the circuit ansatz with respect to *R*_*y*_ gate parameters θ_1_ and θ_2_ for a H–H bond length of 0.7 Å, illustrating the same stationary points in their corresponding colors. The deparameterization procedure by means of setting θ_2_ = 0 corresponds to the horizontal line in red, which intersects with the global minimum. (c) Plot of the absolute difference in energies between transition states and local minima against H–H bond length (logarithmic scale).

The two-qubit *Ĥ*_*q*_ operator of H_2_ in the STO-3G basis consists of five Pauli strings, where H–H bond lengths between 0.1 and 5.0 Å with intervals of 0.1 Å were considered. Utilizing a modified *L* = 1 ansatz equipped with a CNOT gate and a Hartree–Fock initial state of Figure 2, we found that the global minimum was converged to within 10^–10^ hartrees (Ha) of the true ground-state energy for all H–H bond lengths sampled. However, a local minimum was also obtained for each case. We used OPTIM to locate the transition state between the two minima (see section A of the Supporting Information for the coefficients of Pauli strings and stationary points for all of the H–H bond lengths). For example, with a H–H bond length of 0.7 Å, the stationary points can be organized into a disconnectivity graph (Figure 3a), which corresponds to the actual electronic energy landscape parametrized by θ_1_ and θ_2_ (Figure 3b). The transition states lie closer to the local minimum for all bond lengths, as expected from the Hammond postulate,^47^ which can be explained using catastrophe theory.^48^ The difference between the energy of the transition state and the local minimum peaks at a H–H bond length of approximately 1.4 Å before decreasing as the bond length increases further (Figure 3).

The deparameterization procedure may be employed to further simplify the circuit ansatz in Figure 2 by freezing the *R*_*y*_ gate on the second qubit and setting θ_2_ = 0. Using again the illustrative example of 0.7 Å, the result can be visualized by taking a horizontal slice at θ_2_ = 0 in Figure 3b, where the slice intersects with the global minimum. The same circuit ansatz was also successfully employed to obtain only the global minimum for all sampled bond lengths, thus constructing the simplest parameterized circuit ansatz with which to estimate the ground-state energy of H_2_ in the STO-3G basis.

### 3.2 Lithium Hydride

**Figure 4:**
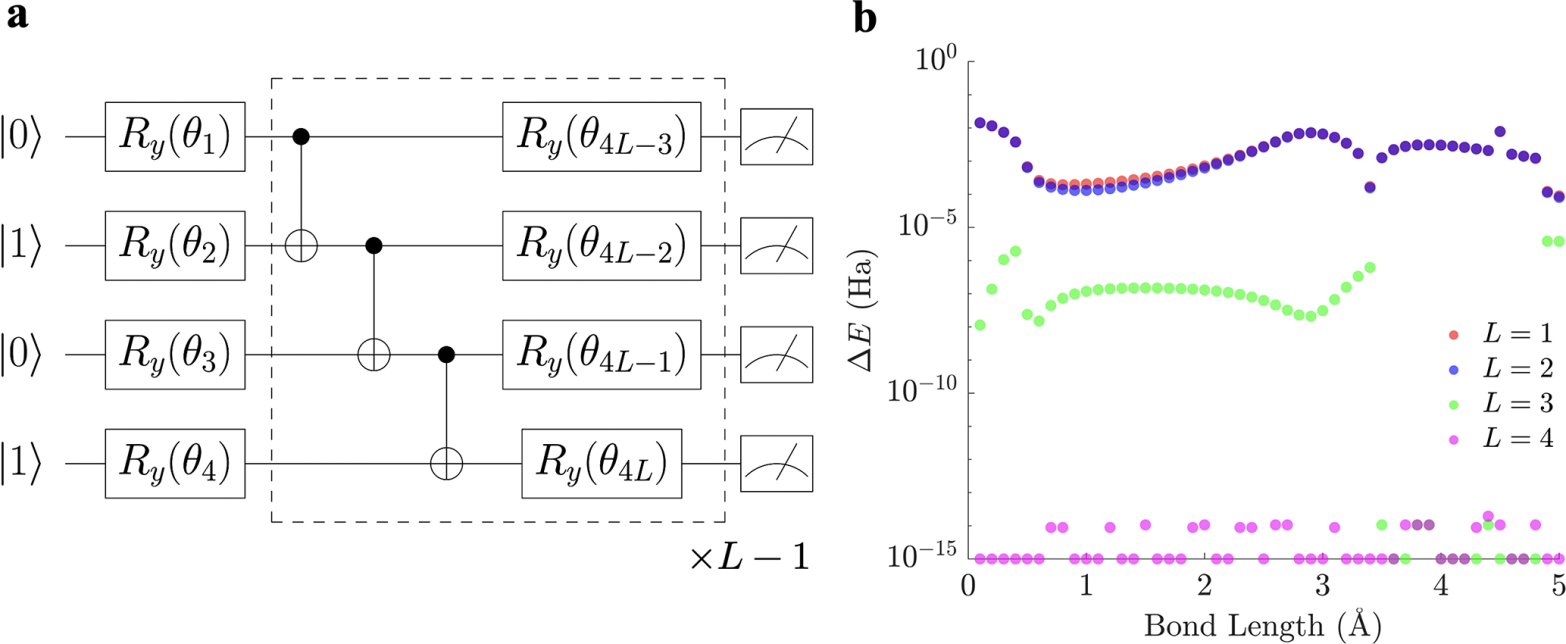
(a) Four-qubit circuit ansatz of LiH used in calculating its ground-state energy. (b) Plot of lowest minima relative to the lowest eigenvalue of the *Ĥ*_*q*_ operator of LiH obtained for various circuit layers *L* across chosen Li–H bond lengths (logarithmic scale).

The *Ĥ*_*q*_ operator for lithium hydride in the STO-3G basis encapsulates two filled orbitals and the next unfilled orbital, thus allowing *Ĥ*_*q*_ to be expressed in a four-qubit form with a linear combination of 100 Pauli strings. Similar to the previous example, Li–H bond lengths between 0.1 and 5.0 Å with intervals of 0.1 Å were considered. Using a Hartree–Fock initial state of  and a corresponding four-qubit *R*_*y*_ ansatz of varying circuit depths (Figure 4a), we found that an *L*_min_ of 4 was required for the global minimum energy to approach within 10^–10^ Ha of the true ground-state energy for all of the Li–H bond lengths considered (Figure 4b). This result illustrates the requirement for a more intricate circuit ansatz to estimate the lowest eigenvalue with a more complex *Ĥ*_*q*_.

**Figure 5:**
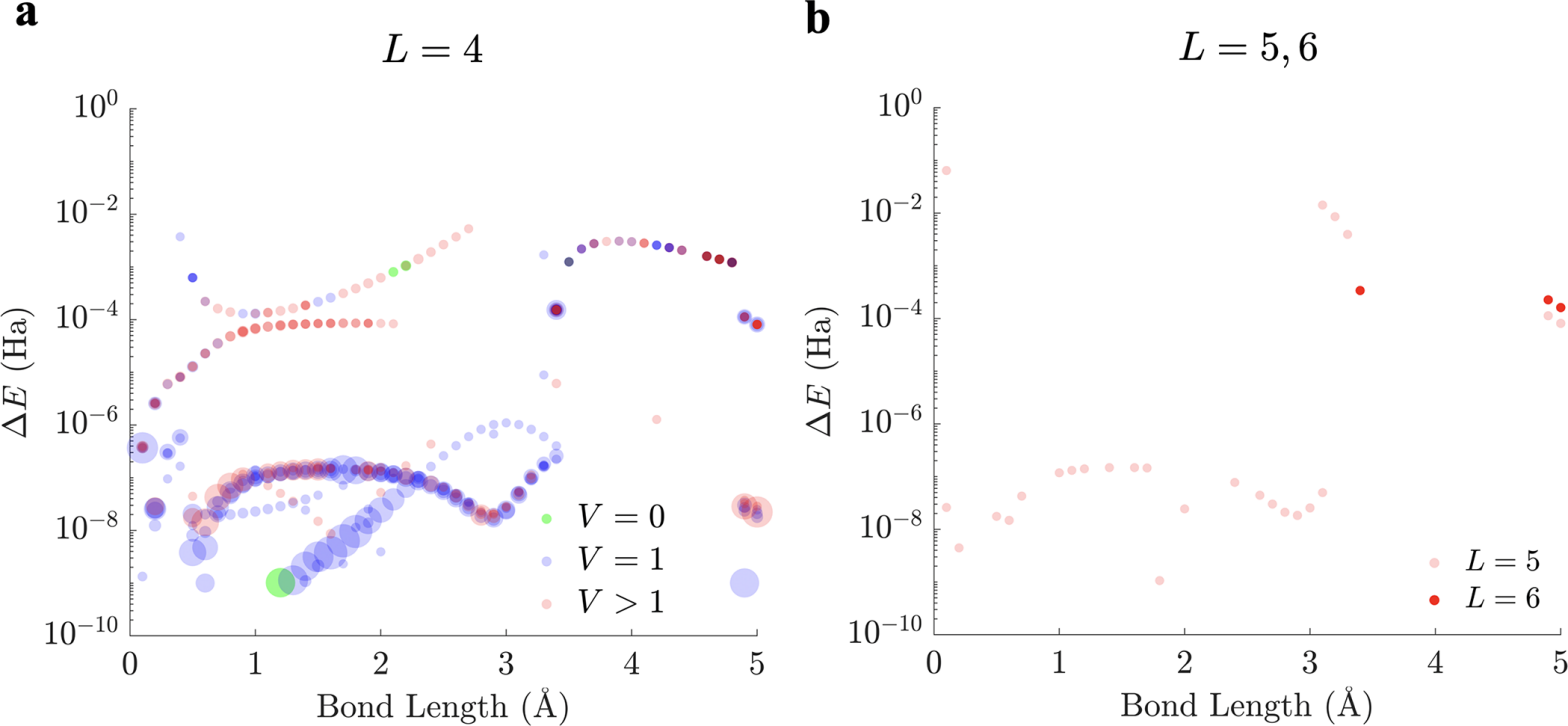
(a) Scatter plot of stationary point energies relative to the lowest eigenvalue of the four-qubit *Ĥ*_*q*_ operator of LiH for *L* = 4 across sampled Li–H bond lengths. Stationary points are further classified into local minima (green), transition states (blue), and high-order saddle points (red) based on the number of negative Hessian eigenvalues *V*. The size of each stationary point indicates its relative frequency for solutions with varying Hessian indexes. (b) Corresponding scatter plot for *L* = 5 (in light red) and *L* = 6 (in dark red), illustrating the drastic reduction in the number of stationary points obtained for both cases. All stationary points obtained are higher order saddle points. Both panels employ logarithmic scales.

An interesting phenomenon occurs when quantum circuits with *L* > *L*_min_ are employed. For the chosen energy convergence criterion of 10^–10^ Ha, the number of other stationary points obtained for *L* = 5 and 6 is much smaller than for *L* = 4 (Figure 5a and 5b). This result suggests that locating the global minimum may be easier for circuits with *L* > *L*_min_, owing to their ability to access more superposition states within the same Hilbert space as *L* = *L*_min_, in contrast to the increasing number of stationary points as the circuit depth increases observed for *L* ≤ *L*_min_. When global optimization benchmarking was carried out for different values of *L* across all Li–H bond lengths using a single core of an Intel Xeon Gold 6248 2.50 GHz CPU (Table 1), we found that the simpler energy landscapes obtained for circuit ansätze with *L* > *L*_min_ outweigh the increase in computation cost normally associated with optimizing a function that depends on more parameters (see section B of the Supporting Information for the optimization times across all sampled Li–H bond lengths). Although the deparameterization procedure for circuits with *L* = 4 provided an improvement in optimization times, the results are not competitive with ansätze for *L* > *L*_min_. This observation can be attributed to the more limited deparameterization for lithium hydride, where only one gate can be frozen if we wish to maintain the accuracy of the global minimum across all Li–H bond lengths. Thus, from an energy landscape perspective, if quantum gate noise is not a significant factor then choosing a circuit ansatz with a depth greater than *L*_min_ may be advantageous in computing the ground-state energy in this example.

**Table 1:** Average CPU Time Taken for a Local Minimization via GMIN To Reach the RMS Gradient Convergence Condition of 5 × 10^–10^ au for Various Circuit Depths *L* Averaged over All Selected Bond Lengths of LiH

*L*	average time to reach RMS convergence, *t* (s)
1	0.0015
2	0.037
3	0.75
4	1.27
4, θ_2_ = 0	0.73
5	0.48
6	0.15

**Figure 6:**
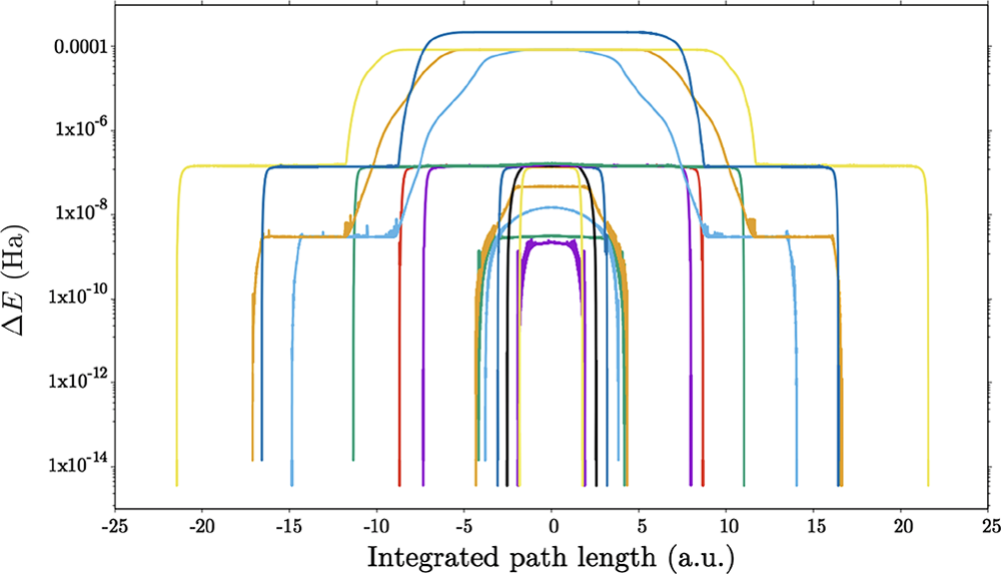
Pathways calculated for each saddle point at an Li–H bond length of 1.5 Å (logarithmic scale). The energy zero is defined by the lowest eigenvalue of the four-qubit *Ĥ*_*q*_ operator of LiH.

For stationary points obtained in the *L* = 4 circuit ansatz, we also observe fewer local minima relative to their corresponding transition states and higher order saddle points. To explore this phenomenon further, we considered the Li–H bond length of 1.5 Å more systematically. Using OPTIM, each stationary point was converged to a tighter convergence RMS gradient criterion of 10^–12^ au, and the pathways between local minima were analyzed for each transition state (Figure 6). We included pathways computed for higher index saddle points, which may be attracted to transition states before converging to the global minimum.

### 3.3 Beryllium Hydride

**Figure 7:**
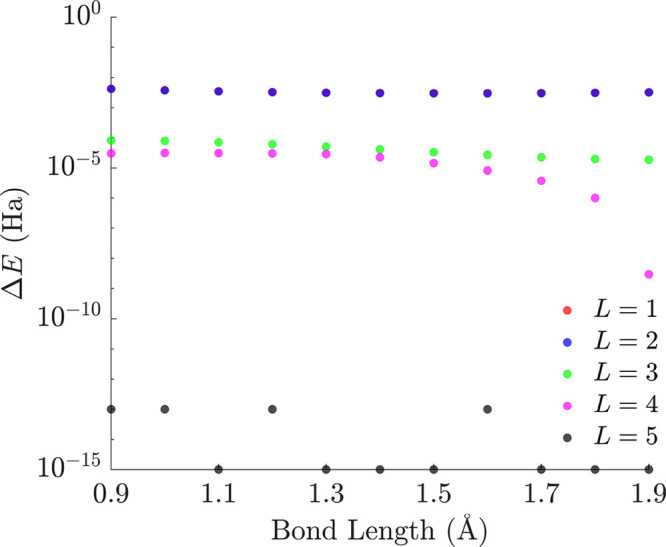
Energy of lowest minima obtained by basin-hopping relative to the lowest eigenvalue of the *Ĥ*_*q*_ operator of BeH_2_ for various circuit layers *L* as a function of the Be–H bond length (logarithmic scale).

**Figure 8:**
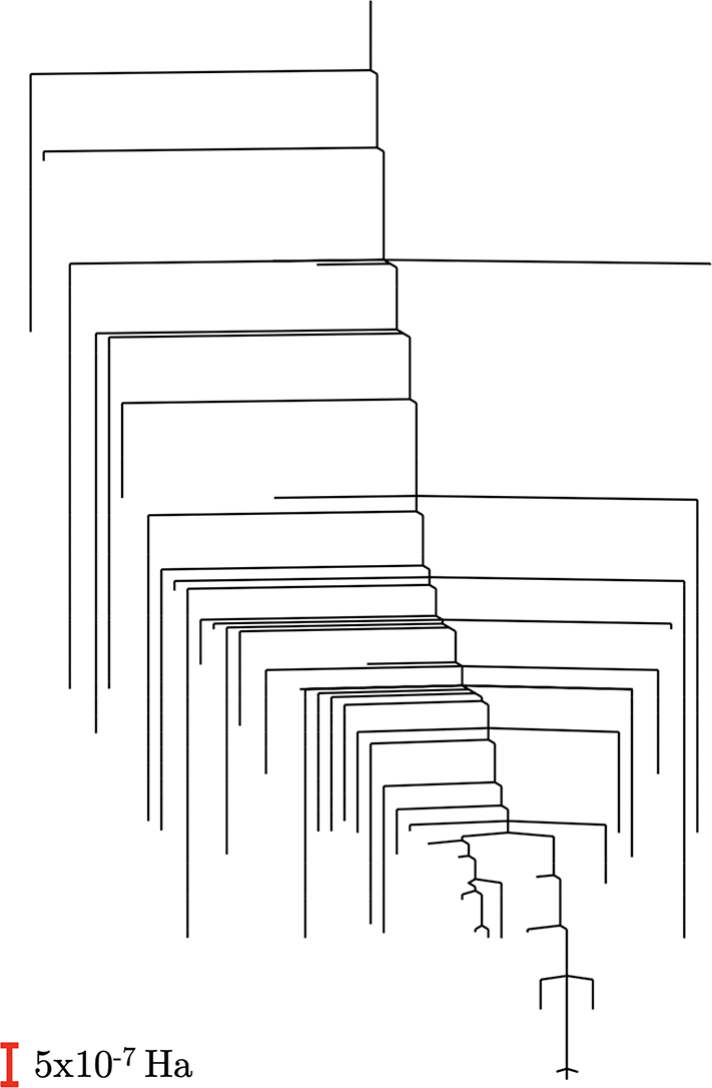
Disconnectivity graph for the *L* = 5 circuit ansatz with Be–H at a bond length of 1.3 Å, including stationary points with an energy below −19.02432 Ha.

The three occupied orbitals and the lowest unoccupied orbital of BeH_2_ in the STO-3G basis were used as the active space, corresponding to a six-qubit *Ĥ*_*q*_ operator. We performed global optimization for *Ĥ*_*q*_ at Be–H bond lengths between 0.9 and 1.9 Å with 0.1 Å intervals using a six-qubit *R*_*y*_ circuit ansatz of varying circuit depths and a Hartree–Fock initial state of  A circuit depth *L*_min_ of 5 was necessary to converge the global minimum energy to within 10^–10^ Ha of the true ground-state value (Figure 7), with the subsequent *L* = 6 ansatz able to reach the same global minimum but requiring a significantly higher computation time to reach convergence (see Table 2) Thus, the *L* = 5 six-qubit *R*_*y*_ circuit ansatz will be the focus of our further analysis for BeH_2_ described below. A key difference between this circuit ansatz and the *L* = 4 LiH results is the presence of additional local minima at every bond length considered. We subsequently selected a Be–H bond length of 1.3 Å to construct a systematic database of local minima and the transition states that connect them and successfully generated the disconnectivity graph for stationary points with energies lower than −19.02432 Ha (Figure 8) or 1.394 × 10^–4^ Ha from the global minimum, thus yielding an illustrative visualization of the energy landscape of the *L* = 5 circuit ansatz with good precision.

**Table 2:** Average CPU Time Taken for a Local Minimization via GMIN To Reach the RMS Gradient Convergence Condition of 5 × 10^–10^ au for Various Circuit Depths *L* Averaged over All Selected Bond Lengths of BeH_2_

*L*	average time to reach RMS convergence, *t* (s)
1	0.026
2	0.077
3	0.666
4	2.77
5	11.4
6	43.0
5, refined	0.18

**Figure 9:**
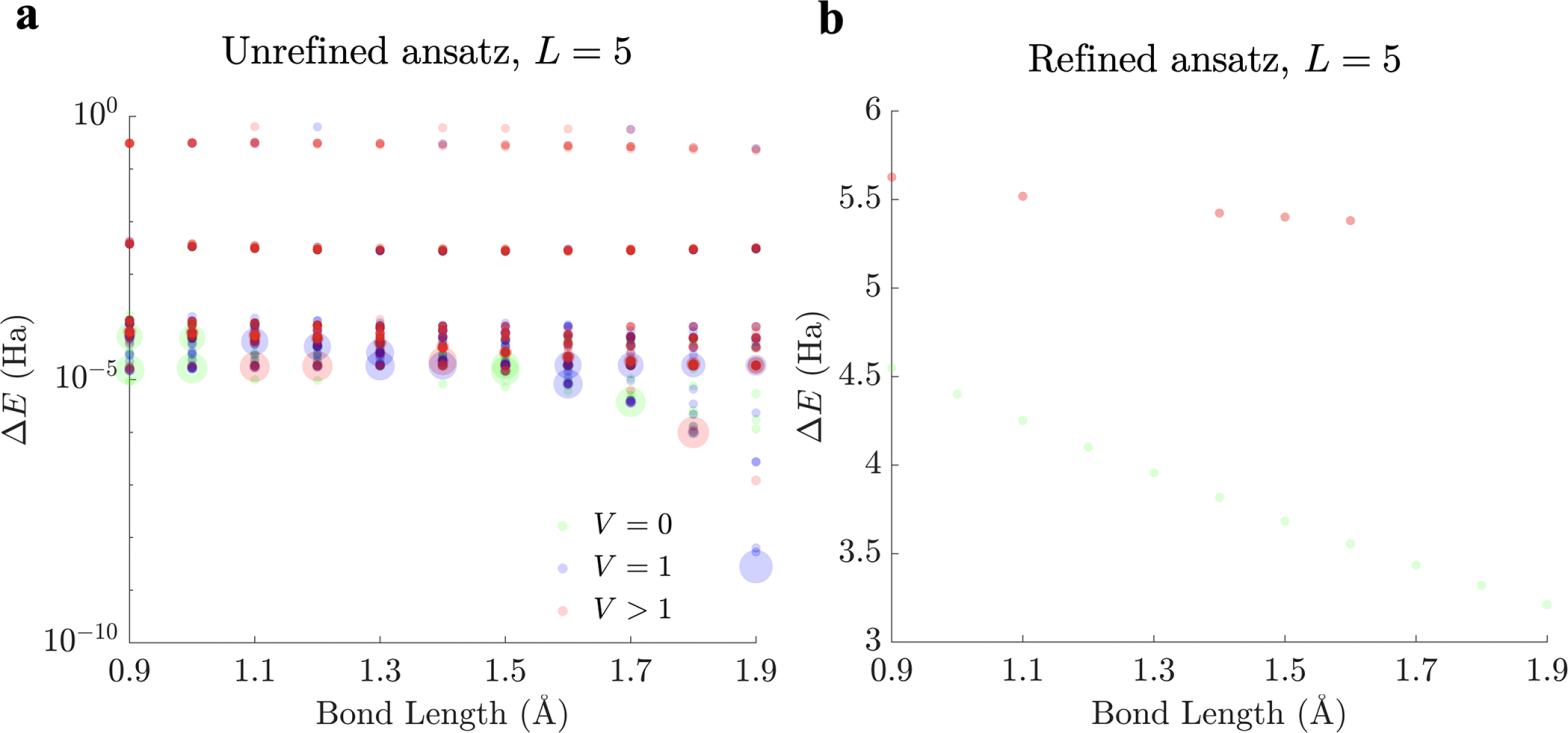
(a) Scatter plot of stationary point energies relative to the lowest eigenvalue of the six-qubit *Ĥ*_*q*_ operator of BeH_2_ for *L* = 5 across sampled Be–H bond lengths. Stationary points are further classified into local minima (green), transition states (blue), and higher order saddle points (red) based on the number of negative Hessian eigenvalues *V*. The size of each stationary point indicates its relative frequency for solutions with varying Hessian indexes. (b) Corresponding scatter plot for the *L* = 5 ansatz simplified with the deparameterization procedure, as in Figure 10, illustrating the significant reduction in the number of stationary points. Both panels employ logarithmic scales.

**Figure 10:**
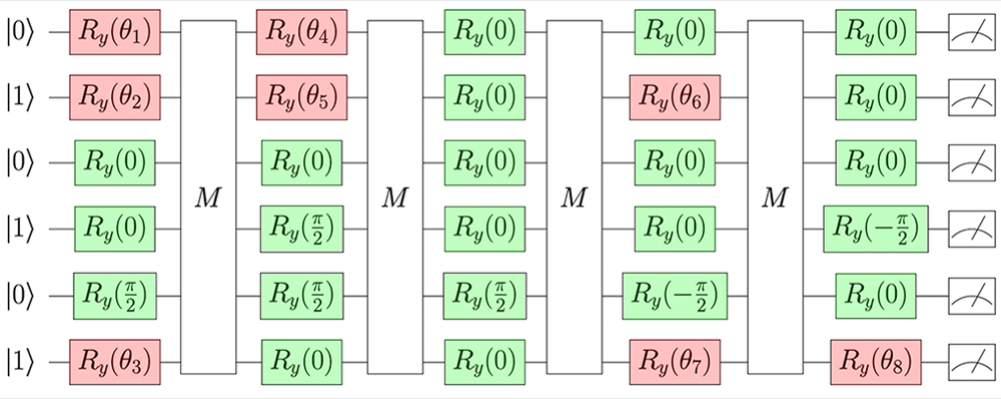
Six-qubit *L* = 5 circuit ansatz of BeH_2_ refined with the deparameterization procedure, illustrating the frozen *R*_*y*_ gates in green with their fixed angles and the retained parametrized *R*_*y*_ gates in red.

We also found that many of the angles in the global minima across all bond lengths tended to the standardized values of {0, ±π/2, ±π}. In particular, for the first run of the deparameterization procedure with the designation of multiple parametrized and deparameterized rotation gates, we froze θ_12_, θ_18_, and θ_29_ while keeping θ_1_, θ_6_, and θ_30_ active (see section D of the Supporting Information for the individual breakdown of rotation coordinates obtained). We then repeated the procedure until we reached the maximum reduction of the number of active parameters from 30 to 8 (Figure 10). This parameter reduction is partially attributable to the relative sparsity of the objective operator *Ĥ*_*q*_ of BeH_2_ compared to LiH: despite its six-qubit form, the number of Pauli string terms is only 95 for the Be–H bond lengths considered, somewhat fewer than that for LiH. The energetic distribution of the stationary points also exhibits a pattern for BeH_2_, with three distinct sets around ∼0.3, ∼0.003, and <0.0001 Ha above the global minimum (Figure 9a). This structure suggests that freezing gate parameters may have a more uniform effect in simplifying the energy landscape. When the energy differences of the other stationary points relative to the global minimum for the refined ansatz were compared to the original ansatz, no examples within the energy bracket of the original ansatz were found. Over the full range of selected bond lengths we located only one new local minimum and at most one higher order saddle point >3 Ha from the global minimum (Figure 9b), suggesting that these new solutions arose from the truncation of the parameter space. Stationary points above the global minimum for the refined ansatz have a significant initial RMS gradient if they are relaxed in the full parameter space. However, since there are fewer of them and the local minima are high in energy, these solutions might be readily identified and discarded even in the presence of significant gate noise from an actual quantum simulator.

The major simplification of the *L* = 5 circuit ansatz energy landscape via the deparameterization procedure, coupled with the reduction in computation cost associated with optimizing fewer parameters, has a significant impact in the time taken for global optimization. We benchmarked this effect for BeH_2_ circuit ansätze with varying layer depth over the full range of Be–H bond lengths considered using a single core of an Intel Xeon Gold 6248 2.50 GHz CPU (Table 2) and found that employing the deparameterization procedure on the *L* = 5 ansatz reduces the average computational cost for a single minimization by a factor of up to 73 (see section C of the Supporting Information for details of the optimization times at all the Be–H bond lengths).

## 4 Conclusion

We have employed the energy landscape framework to explore the solution space of hardware-efficient ansätze with varying circuit depth for H_2_, LiH, and BeH_2_ via the VQE algorithm. The use of basin-hopping methods has provided a platform to largely bypass the obstacle of barren plateaux associated with variational quantum algorithms, enabling us to obtain local minima and other stationary points for which we can reconstruct the energy landscape of the circuit ansatz by means of disconnectivity graphs, as we have demonstrated for BeH_2_ in particular. Characterizing the landscape also enables us to understand the efficiency with which the global minimum can be located, complementing established descriptors such as the innate expressibility or entangling capability of the ansatz.^49^ This insight is especially important for LiH, where although the circuit ansatz of depth *L*_min_ supports a sufficiently accurate global minimum energy, it was not the best choice due to an abundance of alternative stationary points that hinder global optimization. Hence, it may be more efficient to choose a circuit ansatz with a few more layers to access more states within the same Hilbert space, thus bypassing alternative solutions more effectively. For sparser Hamiltonians, we have also tested a deparameterization procedure to freeze redundant *R*_*y*_ gates, which can simplify the energy landscape while retaining the accuracy of the global minimum. For BeH_2_, we find that deparameterization significantly reduces the computational expense of global optimization. The reduction of parametrized gates to virtual identity gates is also expected to be useful in reducing the noise attributed to practical implementation of quantum gates as well as more efficient circuit transpilation, thus providing tangible benefits to both hardware and software aspects. Exploiting quantum computing to profile molecular energy landscapes is an exciting prospect, and we aim to use the methods developed here to explore circuit ansätze for other variational algorithms such as UCC in future work. Finally, we also envision that our methods can be utilized in other variational quantum algorithms where layer-based ansätze are readily employed, for example in the Quantum Approximate Optimization Algorithm (QAOA) in solving combinatorial optimization problems,^50^ as well as quantum neural networks in trainable quantum machine learning models.^51^

## Data Availability

The data that support the findings of this study are available within the article and the Supporting Information provided.

## Supporting Information Available

(ZIP)
(PDF)
