# Supplementary figures and images for: Molecular Energy Landscapes of Hardware-Efficient Ansätze in Quantum Computing
Source: J Chem Theory Comput. 2023 Feb 7;19(4):1197–206. doi: 10.1021/acs.jctc.2c01057 (PMC9979602; doi:10.1021/acs.jctc.2c01057)

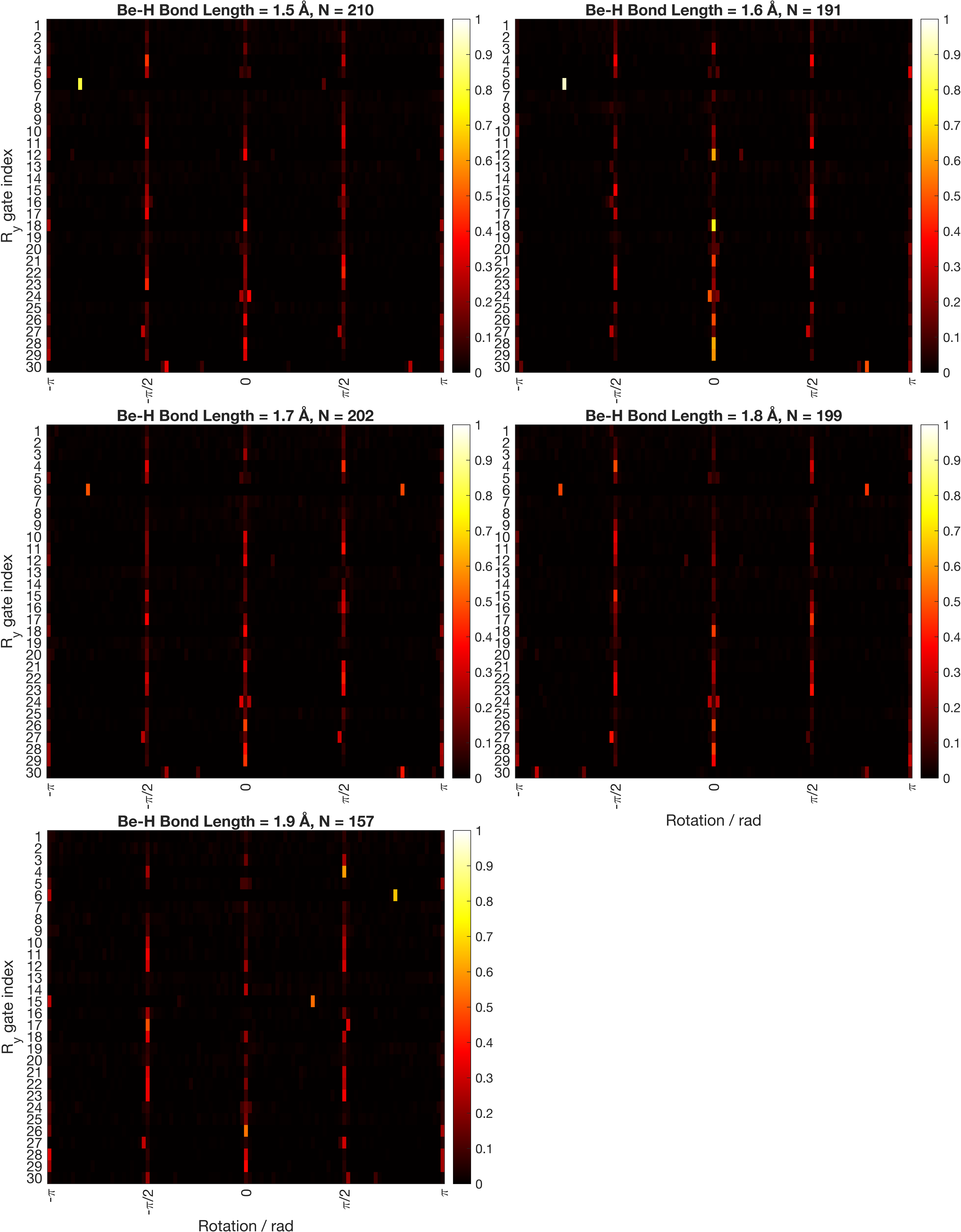

Supplement: Supplementary file 1 — ct2c01057_si_001.zip [file ct2c01057_si_001.zip › BeH2_A2.png]

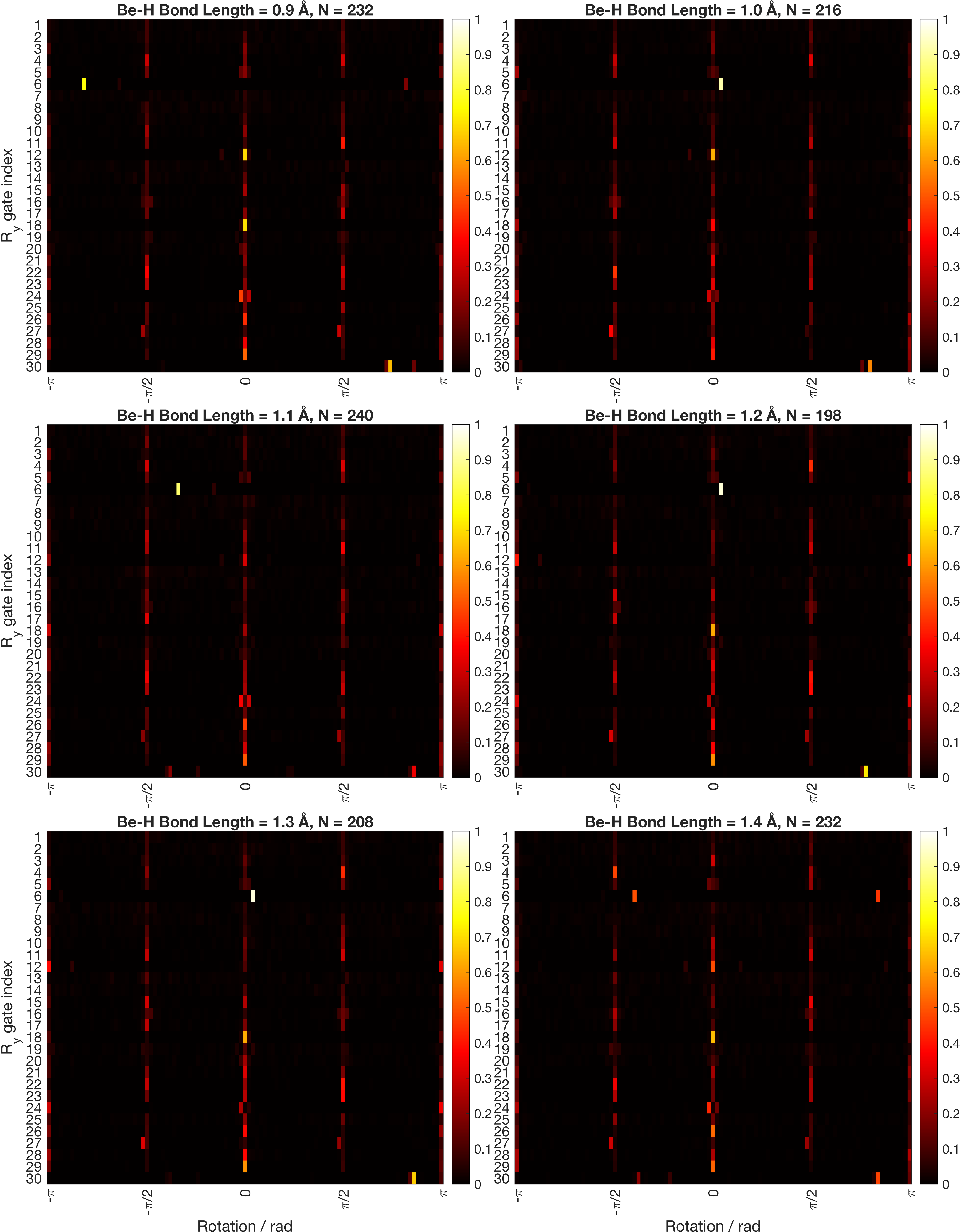

Supplement: Supplementary file 1 — ct2c01057_si_001.zip [file ct2c01057_si_001.zip › BeH2_A1.png]
