# Supplementary material for: Molecular Energy Landscapes of Hardware-Efficient Ansätze in Quantum Computing
Source: J Chem Theory Comput. 2023 Feb 7;19(4):1197–206. doi: 10.1021/acs.jctc.2c01057 (PMC9979602; doi:10.1021/acs.jctc.2c01057)
Supplement: Supplementary file 2 — ct2c01057_si_002.pdf [file ct2c01057_si_002.pdf]

Supplementary Material: Molecular Energy Landscapes of Hardware-efficient Ansätze in Quantum Computing

Choy Boy[1,a] and David J. Wales[2]

[1]School of Chemistry, Chemical Engineering and Biotechnology, Nanyang Technological University, Block N1.2, B3-13, 62 Nanyang Drive, Singapore 637459

[2]Yusuf Hamied Department of Chemistry, University of Cambridge, Lensfield Road, Cambridge, CB2 1EW, U.K.

[a]Electronic mail: CHOY0065@e.ntu.edu.sg

Section 1: Optimisation Regime in Estimating the Ground-state Energy via GMIN

For each individual bond length of a given molecule, 5000 basin-hopping iterations were carried out via the GMIN package. All gradients for each parameterised rotation gate were calculated with the parameter-shift rule. Each iteration has a root-mean squared (RMS) gradient convergence criterion of 1.0×10^{-10} a.u. to proceed to the next run, following which a random perturbation of up to 1.0 rad is carried out for each rotation parameter of the starting coordinates for the following run. If the current basin-hopping run attains a tighter RMS gradient convergence tolerance of 5.0×10^{-10} a.u., its respective energy and angular coordinates will be saved. The maximum number of iterations allowed for each LBFGS minimisation run is set at 100000. For each iteration step j, the change in energy will automatically be accepted if its corresponding energy E_j is greater in energy than the subsequent iteration, i.e. $E_j > E_{j+1}$. If $E_j \leq E_{j+1}$, then E_{j+1} will be accepted with probability equal to $\exp[-(E_{j+1} - E_j)/k_BT]$, where the reduced temperature k_BT is set at 1.0 a.u. Otherwise, if E_{j+1} is rejected, a random perturbation of up to 1.0 rad is carried out for each rotation parameter and a new energy obtained. At the end of the GMIN regime, quenched stationary points with energies and their corresponding rotation parameters that differ by at least 1.0×10^{-9} Ha are collated and compiled.

Section A: Coefficients of Pauli strings and stationary points of H_2

H−H Bond Length (Å)	I_1I_2	Z_1I_2 ($-I_1Z_2$)	Z_1Z_2	X_1X_2	Global minimum (Ha)	Local minimum (Ha)	Transition state (Ha)
0.1	-0.5351220074	-1.0273146838	-0.0138666592	0.1561702591	2.7099607709	4.5866131835	4.5866274109
0.2	-0.6343578506	-0.9304885285	-0.0136238651	0.1579727086	0.1574821348	1.8399316302	1.8399485640
0.3	-0.7537419522	-0.8086489099	-0.0132879771	0.1608185192	-0.6018037108	0.8360755879	0.8360973016
0.4	-0.8625795317	-0.6888194296	-0.0129139693	0.1645154240	-0.9141497046	0.2829341023	0.2829630173
0.5	-0.9477078770	-0.5830796255	-0.0125164316	0.1688702277	-1.0551597945	-0.0707401144	-0.0707012029
0.6	-1.0071270770	-0.4940137866	-0.0120643897	0.1737306437	-1.1162860069	-0.3109600923	-0.3109082787
0.7	-1.0439125218	-0.4204556798	-0.0115074022	0.1790005761	-1.1361894541	-0.4784530558	-0.4783860026
0.8	-1.0632127971	-0.3599594245	-0.0108097350	0.1846267836	-1.1341476667	-0.5971778020	-0.5970945325
0.9	-1.0702832691	-0.3097872795	-0.0099691083	0.1905716938	-1.1205602813	-0.6828493924	-0.6827506903
1.0	-1.0692434904	-0.2675286499	-0.0090149301	0.1967905835	-1.1011503302	-0.7458717930	-0.7457600342
1.1	-1.0628124875	-0.2313958771	-0.0079951751	0.20322222662	-1.0791929450	-0.7929596975	-0.7928383542
1.2	-1.0526707246	-0.2001895768	-0.0069621624	0.20979146862	-1.0567407463	-0.8284433465	-0.8283164369
1.3	-1.0399165885	-0.1731078530	-0.0059622856	0.21641745962	-1.0351862664	-0.8552369408	-0.8551085354
1.4	-1.0253590356	-0.1495679388	-0.0050305364	0.22302208907	-1.0154682493	-0.8754279390	-0.8753017585
1.5	-1.0096446944	-0.1291013129	-0.0041889583	0.22953593606	-0.9981493535	-0.8905847814	-0.8904639205
1.6	-0.9932985016	-0.1113087473	-0.0034477894	0.23590128540	-0.9834727290	-0.9019118196	-0.9017986245
1.7	-0.9767372365	-0.0958445867	-0.0028080706	0.24207283852	-0.9714266885	-0.9103374333	-0.9102335155
1.8	-0.9602805783	-0.0824097916	-0.0022646741	0.24801699348	-0.9618169528	-0.9165749065	-0.9164812393
1.9	-0.9441646083	-0.0707457885	-0.0018090188	0.25371042781	-0.9543388540	-0.9211697333	-0.9210867742
2.0	-0.9285563459	-0.0606280089	-0.0014311039	0.25913847488	-0.9486411122	-0.9245373192	–0.9244651157
2.1	-0.9135674705	-0.0518600664	-0.0011208035	0.26429356608	-0.9443746811	-0.9269926920	-0.9269309615
2.2	-0.8992663202	-0.0442693402	-0.0008685547	0.26917385594	-0.9412240337	-0.9287736350	-0.9287218227
2.3	-0.8856879934	-0.0377040077	-0.0006656392	0.27378205414	-0.9389223860	-0.9300586385	-0.9300159696

H−H Bond Length (Å)	$I_1 I_2$	$Z_1 I_2$ ($-I_1 Z_2$)	$Z_1 Z_2$	$X_1 X_2$	Global minimum (Ha)	Local minimum (Ha)	Transition state (Ha)
2.4	-0.8728426912	-0.0320310581	-0.0005042423	0.27812444315	-0.9372549530	-0.9309808721	-0.9309464076
2.5	-0.8607225052	-0.0271346994	-0.0003774199	0.28221004598	-0.9360549200	-0.9316390867	-0.9316117867
2.6	-0.8493068403	-0.0229147310	-0.0002790445	0.28604991175	-0.9351960308	-0.9321061000	-0.9320848898
2.7	-0.8385666490	-0.0192847148	-0.0002037583	0.28965649669	-0.9345844159	-0.9324353444	-0.9324191759
2.8	-0.8284676561	-0.0161700000	-0.0001469354	0.29304312867	-0.9341510957	-0.9326658592	-0.9326537604
2.9	-0.8189727542	-0.0135057666	-0.0001046455	0.29622355084	-0.9338457508	-0.9328260503	-0.9328171583
3.0	-0.8100437434	-0.0112352608	-0.0000736103	0.29921154326	-0.9336318446	-0.9329364933	-0.9329300714
3.1	-0.8016425676	-0.0093083417	-0.0000511489	0.30202062153	-0.9334829404	-0.9330120119	-0.9330074522
3.2	-0.7937321721	-0.0076803756	-0.0000351139	0.30466380888	-0.9333799773	-0.9330632164	-0.9330600323
3.3	-0.7862770781	-0.0063114528	-0.0000238195	0.30715347580	-0.9333092724	-0.9330976427	-0.9330954553
3.4	-0.7792437458	-0.0051658558	-0.0000159682	0.30950123844	-0.9332610575	-0.9331205962	-0.9331191176
3.5	-0.7726007791	-0.0042117001	-0.0000105804	0.31171790574	-0.9332284056	-0.9331357764	-0.9331347928
3.6	-0.7663190124	-0.0034206725	-0.0000069296	0.31381346482	-0.9332064420	-0.9331457371	-0.9331450932
3.7	-0.7603715105	-0.0027678087	-0.0000044865	0.31579709468	-0.9331917641	-0.9331522239	-0.9331518090
3.8	-0.7547335074	-0.0022312767	-0.0000028716	0.31767719949	-0.9331820168	-0.9331564177	-0.9331561546
3.9	-0.7493823031	-0.0017921457	-0.0000018170	0.31946145437	-0.9331755831	-0.9331591101	-0.9331589459
4.0	-0.7442971350	-0.0014341392	-0.0000011365	0.32115685795	-0.9331713618	-0.9331608268	-0.9331607259
4.1	-0.7394590360	-0.0011433732	-0.0000007028	0.32276978752	-0.9331686087	-0.9331619138	-0.9331618529
4.2	-0.7348506879	-0.0009080919	-0.0000004296	0.32430605411	-0.9331668238	-0.9331625976	-0.9331625613
4.3	-0.7304562774	-0.0007184059	-0.0000002596	0.32577095521	-0.9331656739	-0.9331630246	-0.9331630033
4.4	-0.7262613578	-0.0005660441	-0.0000001550	0.32716932439	-0.9331649379	-0.9331632893	-0.9331632770
4.5	-0.7222527195	-0.0004441237	-0.0000000915	0.32850557695	-0.9331644701	-0.9331634522	-0.9331634452
4.6	-0.7184182709	-0.0003469426	-0.0000000534	0.32978375149	-0.9331641749	-0.9331635517	-0.9331635478
4.7	-0.7147469296	-0.0002697949	-0.0000000308	0.33100754748	-0.9331639901	-0.9331636119	-0.9331636097
4.8	-0.7112285238	-0.0002088109	-0.0000000175	0.33218035895	-0.9331638754	-0.9331636480	-0.9331636468
4.9	-0.7078537041	-0.0001608188	-0.0000000099	0.33330530464	-0.9331638049	-0.9331636694	-0.9331636688
5.0	-0.7046138639	-0.0001232276	-0.0000000055	0.33438525484	-0.9331637619	-0.9331636821	-0.9331636817

Section B: Basin-hopping global optimisation times of LiH for varying circuit depths

Li−H Bond Length (Å)	Average time to reach convergence for 5000 basin-hopping steps / s						
	$L=1$	$L=2$	$L=3$	$L=4$	$L=4, \theta_2=0$	$L=5$	$L=6$
0.1	0.0018	0.0656	2.1274	2.1864	1.5670	0.4988	0.0926
0.2	0.0018	0.0554	1.2182	2.7216	1.0308	0.4098	0.0990
0.3	0.0016	0.0568	0.9884	2.9466	0.6780	0.3258	0.0988
0.4	0.0018	0.0074	0.9574	2.9958	0.6018	0.2940	0.0960
0.5	0.0018	0.0072	1.0274	1.8610	0.8744	0.3684	0.1024
0.6	0.0014	0.0068	0.7446	1.9486	1.0502	0.4768	0.1028
0.7	0.0016	0.0068	1.0162	1.6536	1.0502	0.4650	0.1006
0.8	0.0016	0.0072	1.2250	1.6872	1.0798	0.4634	0.1020
0.9	0.0016	0.0072	1.5774	1.7388	1.0810	0.4628	0.0992
1.0	0.0016	0.0072	1.8086	1.4598	1.0036	0.4542	0.0972
1.1	0.0016	0.0072	1.7616	1.4558	0.9798	0.4390	0.0964
1.2	0.0016	0.0060	1.5586	1.4872	0.9778	0.4494	0.0954
1.3	0.0016	0.0062	1.4288	1.5242	0.9676	0.4412	0.0952
1.4	0.0016	0.0062	1.3162	1.5682	0.9884	0.4488	0.0904
1.5	0.0014	0.0062	1.1230	1.3588	0.9856	0.4502	0.0996
1.6	0.0014	0.0064	1.0004	1.6174	1.0128	0.4576	0.0906
1.7	0.0016	0.0062	0.9486	1.5956	0.9570	0.4802	0.0878
1.8	0.0016	0.0064	0.8642	1.5514	1.0410	0.4392	0.0878
1.9	0.0016	0.0046	0.8580	1.5350	1.0124	0.4182	0.0842
2.0	0.0016	0.0064	0.8208	1.4422	0.9262	0.3860	0.0826
2.1	0.0016	0.0064	0.7594	1.3412	0.7922	0.3718	0.0824
2.2	0.0014	0.0064	0.6612	1.3318	0.7444	0.3612	0.0794
2.3	0.0016	0.0078	0.6580	1.3180	0.6736	0.3458	0.0806
2.4	0.0016	0.0062	0.6074	1.1586	0.6232	0.3314	0.0784
2.5	0.0016	0.0076	0.5468	1.0666	0.5800	0.3280	0.0760
2.6	0.0018	0.0064	0.4922	1.0096	0.5482	0.3276	0.0766
2.7	0.0018	0.0094	0.4172	1.0060	0.4974	0.3216	0.0786
2.8	0.0016	0.0468	0.5432	0.9432	0.4768	0.3252	0.0824
2.9	0.0016	0.0588	0.6354	0.8850	0.4910	0.3414	0.0890
3.0	0.0016	0.0848	0.6566	0.9684	0.4572	0.3432	0.0990
3.1	0.0014	0.1036	0.6924	1.0642	0.4888	0.3568	0.1108
3.2	0.0014	0.1040	0.7232	1.2472	0.5402	0.3678	0.1396
3.3	0.0014	0.1036	0.8112	1.4298	0.6586	0.3902	0.3192
3.4	0.0014	0.1116	1.0424	2.2432	1.2468	0.5794	0.2152
3.5	0.0016	0.1176	0.0894	0.3470	0.2658	0.5534	0.1946
3.6	0.0014	0.1014	0.2156	0.5010	0.3946	0.6174	0.1794

Li−H Bond Length (Å)	Average time to reach convergence for 5000 basin-hopping steps / s						
	$L=1$	$L=2$	$L=3$	$L=4$	$L=4, \theta_2=0$	$L=5$	$L=6$
3.7	0.0014	0.0810	0.1586	0.4168	0.3698	0.5960	0.1696
3.8	0.0014	0.0756	0.1384	0.3882	0.3542	0.6014	0.1678
3.9	0.0014	0.0718	0.1268	0.3620	0.3370	0.6572	0.1732
4.0	0.0014	0.0692	0.1196	0.3676	0.3362	0.6352	0.1580
4.1	0.0012	0.0646	0.1222	0.3832	0.3382	0.6284	0.1638
4.2	0.0012	0.0428	0.1176	0.3788	0.3374	0.7070	0.1728
4.3	0.0012	0.0344	0.1202	0.3782	0.3452	0.7024	0.1764
4.4	0.0014	0.0320	0.1208	0.4064	0.3436	0.7042	0.1242
4.5	0.0014	0.0958	0.0998	0.3226	0.2978	0.7330	0.1952
4.6	0.0012	0.0276	0.1244	0.4338	0.3766	0.7002	0.2132
4.7	0.0012	0.0246	0.1226	0.4456	0.3908	0.7074	0.2138
4.8	0.0014	0.0240	0.1306	0.4798	0.3820	0.6870	0.4382
4.9	0.0014	0.0262	1.1224	2.1816	1.4004	0.7596	0.5032
5.0	0.0012	0.0232	0.9850	2.3578	1.4982	0.0948	0.7596

Section C: Basin-hopping global optimisation times of BeH_2 for varying circuit depths

Be−H Bond Length (Å)	Average time to reach convergence for 5000 basin-hopping steps / s						
	$L=1$	$L=2$	$L=3$	$L=4$	$L=5$	$L=6$	$L=5$, refined
0.9	0.0238	0.0774	0.6476	2.6498	11.0324	29.5300	0.1756
1.0	0.0254	0.0768	0.6230	2.7520	10.9068	29.3182	0.1744
1.1	0.0254	0.0764	0.6440	2.7226	11.5552	28.8484	0.1754
1.2	0.0256	0.0760	0.6598	2.7206	12.8740	29.4592	0.1764
1.3	0.0258	0.0774	0.6598	2.7666	12.3582	50.7478	0.1760
1.4	0.0256	0.0766	0.6906	2.7856	11.0878	50.5306	0.1778
1.5	0.0258	0.0768	0.6772	2.7628	11.1754	50.9514	0.1826
1.6	0.0260	0.0776	0.6734	2.9560	11.3072	50.8396	0.1820
1.7	0.0270	0.0764	0.6802	2.8794	10.8224	50.8768	0.1862
1.8	0.0264	0.0766	0.6764	2.6012	12.4404	51.5582	0.1858
1.9	0.0268	0.0772	0.6950	2.9080	9.9344	49.9414	0.1844

Section D: Amplitude values of global minima of the BeH_2 $L = 5$ circuit ansatz

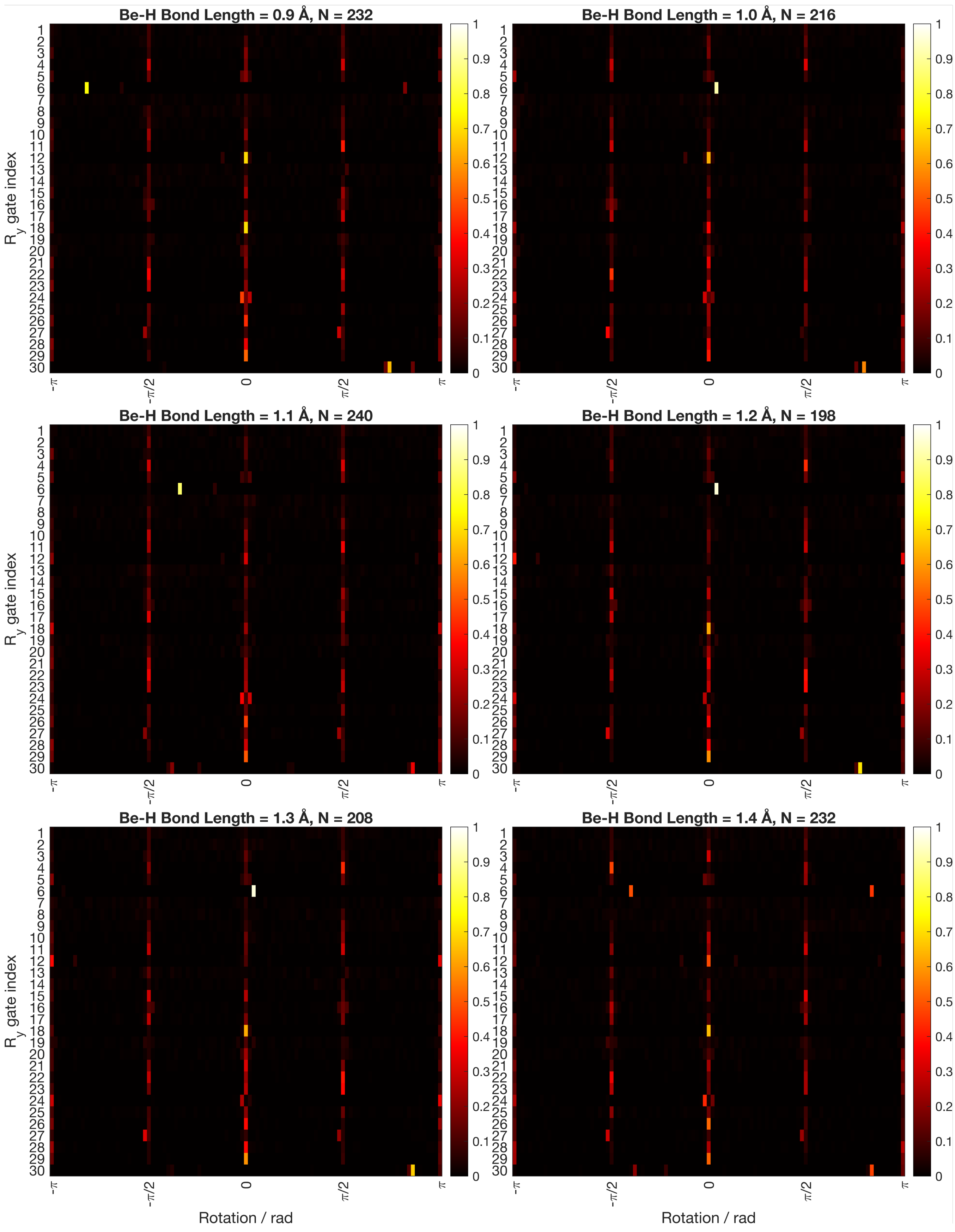

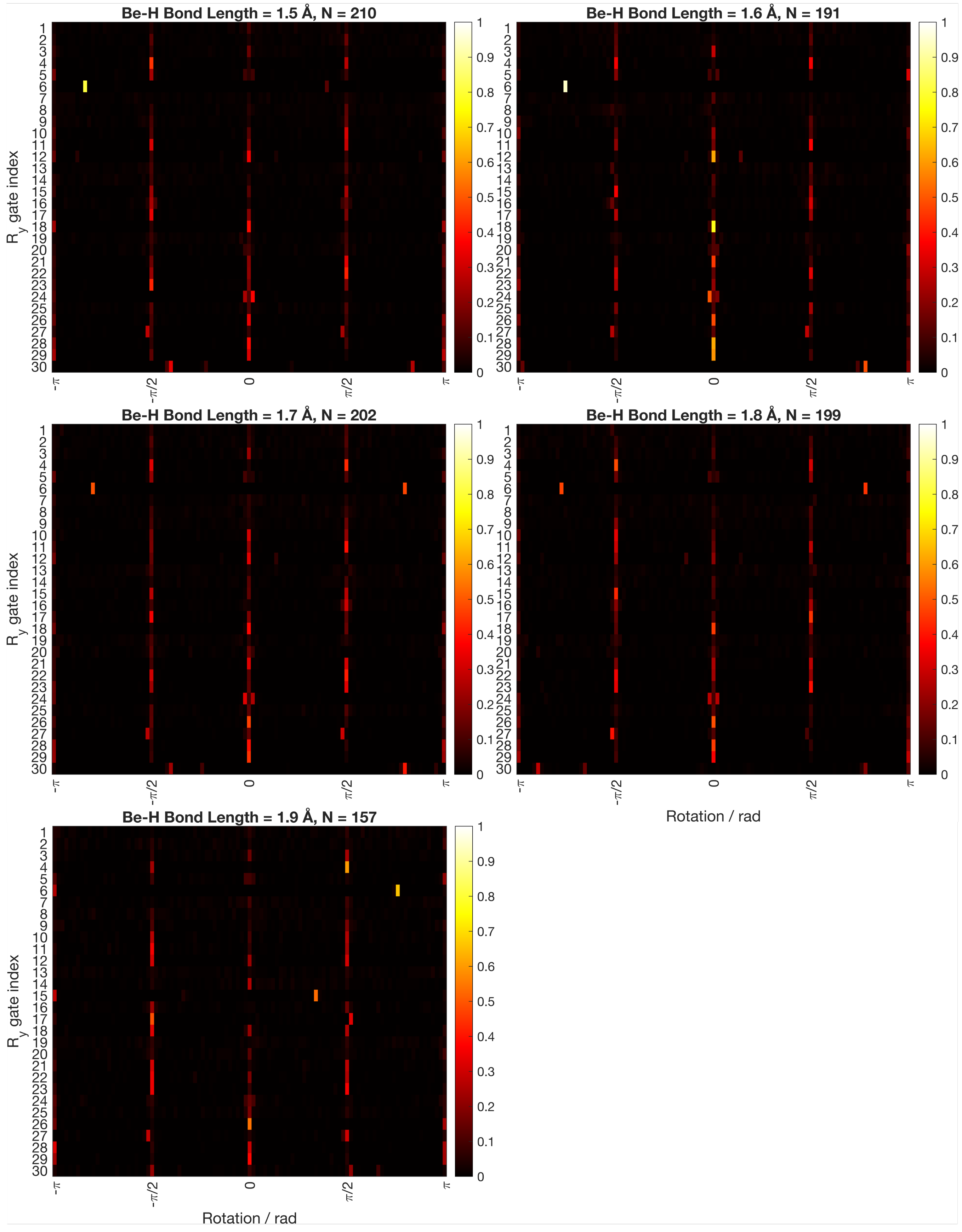

Heatmaps illustrating the rotation amplitudes of individual R_y rotation gates from global minima obtained from GMIN of the BeH_2 $L = 5$ circuit ansatz with varying Be−H bond lengths, as a proportion of the total number of unique coordinates N obtained from 5000 basin-hopping runs via GMIN for each Be−H bond length. For the first run of the deparameterisation procedure, θ_{12}, θ_{18} and θ_{29} were frozen with a rotation value of zero, while θ_1, θ_6 and θ_{30} were designated as gates that should remain parameterised.
